# Blood‐based pre‐screening in the SKYLINE secondary prevention Ph3 gantenerumab study

**DOI:** 10.1002/alz.70676

**Published:** 2025-10-14

**Authors:** Alina Bauer, Christina Rabe, Courtney Schiffman, Fiona Rose, Gesine Respondek, Fabiana Gullotta, Laura Schlieker, Alexander Jethwa, Isabelle Schrurs, Ekaterina Manuilova, Susanne Ostrowitzki, Tobias Bittner

**Affiliations:** ^1^ Roche Diagnostics GmbH Penzberg Germany; ^2^ Genentech, Inc. South San Francisco California USA; ^3^ Roche Products Ltd Welwyn Garden City UK; ^4^ F. Hoffmann‐La Roche Ltd Basel Switzerland; ^5^ Staburo GmbH (on behalf of Roche Diagnostics GmbH) Munich Germany; ^6^ Roche Diagnostics International Ltd Rotkreuz Switzerland

**Keywords:** Alzheimer's disease, amyloid beta 40, amyloid beta 42, apolipoprotein E4 protein, blood‐based biomarkers, gantenerumab, glial fibrillary acidic protein, phosphorylated tau 181, pre‐screening, secondary prevention

## Abstract

**INTRODUCTION:**

SKYLINE was a secondary prevention study that used blood‐based biomarker (BBBM) pre‐screening to screen out participants with a low likelihood of amyloid positivity by positron emission tomography (PET) or cerebrospinal fluid (CSF) testing.

**METHODS:**

This retrospective analysis used data from SKYLINE (ClinicalTrials.gov: NCT05256134; terminated prematurely) and the Anti‐Amyloid Treatment in Asymptomatic Alzheimer's (A4) study to compare predicted and actual clinical performance characteristics of various biomarker combinations using prototype Elecsys^®^ plasma immunoassays (Roche Diagnostics International Ltd, Rotkreuz, Switzerland).

**RESULTS:**

In >3500 participants screened in SKYLINE, tau phosphorylated at threonine 181 (pTau181) and apolipoprotein E4 protein (ApoE4p) was the highest‐performing BBBM combination. Actual clinical performance of the BBBM pre‐screening in SKYLINE was similar to predictions based on A4 in terms of screen‐out rate, positive predictive value, and 1‐negative predictive value.

**DISCUSSION:**

BBBM pre‐screening in SKYLINE using prototype plasma pTau181 and ApoE4p immunoassays effectively alleviated participant burden by avoiding unnecessary PET or CSF testing.

**Highlights:**

- We compared blood‐based biomarker (BBBM) performance in SKYLINE and Anti‐Amyloid Treatment in Asymptomatic Alzheimer's (A4).
- Pre‐screening improved amyloid positivity (defined by positron emission tomography/cerebrospinal fluid) screen failure rate.
- Tau phosphorylated at threonine 181 (pTau181) and apolipoprotein E4 protein was the highest‐performing combination among BBBMs tested.
- Pre‐screening eased participant burden by reducing subsequent screening procedures.

## 1 BACKGROUND

Alzheimer's disease (AD) is a debilitating and progressive neurodegenerative disease that accounts for 60% to 80% of dementia cases in the United States.^1^, ^2^ The prevalence of AD and other dementias is increasing globally, with a projected 152.8 million cases expected by 2050.^3^ AD is neuropathologically defined by the presence of amyloid beta (Aβ) plaques and neurofibrillary tangles containing aggregated hyperphosphorylated tau proteins.^4^, ^5^ Recent biomarker evidence suggests that the underlying amyloid and tau pathologies begin to present 10 to 30 years before the onset of clinical symptoms, which may provide a window of opportunity for preventive therapies to be administered.^6^, ^7^, ^8^

Although anti‐Aβ treatments have recently demonstrated efficacy in symptomatic disease (mild cognitive impairment or mild dementia), it has been hypothesized that these treatments may have a more profound benefit if introduced when AD pathology is present but before the onset of cognitive impairment, that is, in the secondary prevention setting.^9^ The objective of secondary prevention studies is to demonstrate that the onset of clinical symptoms can be slowed or delayed if treatment is administered prior to their onset.^9^ One such study was the Anti‐Amyloid Treatment in Asymptomatic Alzheimer's (A4) study, which tested solanezumab in individuals with preclinical AD to slow down cognitive and functional decline.^10^ More secondary prevention studies are currently underway in AD.^11^, ^12^, ^13^

A key challenge in recruiting participants for secondary prevention trials is that the prevalence of amyloid‐positive individuals with positron emission tomography (PET) visual read and cerebrospinal fluid (CSF) in a population of cognitively unimpaired individuals 60 to 80 years of age was expected to be between 10% and 20% based on internal analysis and the previous literature.^7^, ^14^, ^15^, ^16^ Both methods are burdensome for individuals and clinical sites, particularly if performed on cognitively unimpaired individuals to identify amyloid positivity for secondary prevention studies.^9^, ^17^ Blood‐based biomarkers (BBBMs) are minimally invasive and cost‐effective, and are a potential tool for ruling out a substantial number of individuals with a high likelihood of amyloid negativity. Only individuals who screen positive for BBBM would be referred for the more invasive, costly, and time‐consuming subsequent screening assessments.^17^, ^18^

Gantenerumab is a fully human, anti‐Aβ immunoglobulin G1 monoclonal antibody with high affinity for aggregated Aβ.^19^ The gantenerumab program included a global, multi‐center, double‐blind, placebo‐controlled, Phase 3 secondary prevention study, SKYLINE (NCT05256134). Clinical development of gantenerumab was discontinued,^20^ and, consequently, the SKYLINE study was terminated early. SKYLINE was designed to investigate the efficacy and safety of gantenerumab in cognitively unimpaired individuals either at risk for or at the earliest stages of AD.^13^, ^21^ In order to facilitate recruitment, the study used a BBBM algorithm to rule out presumed amyloid‐negative individuals at pre‐screening, before participants entered the main screening phase. The biomarker combination and algorithm used were established using previously collected samples and datasets from other cohorts, including Biomarkers For Identifying Neurodegenerative Disorders Early and Reliably (BioFINDER),^22^ Australian Imaging, Biomarker & Lifestyle (AIBL),^23^ and A4.^24^

In this retrospective analysis, the predicted (based on prevalence‐adjusted historic A4 cohort data) and actual performance of the BBBM pre‐screening in SKYLINE were compared. In addition, a head‐to‐head performance comparison of the pre‐screening algorithm in the SKYLINE and A4 cohorts was carried out. The impact of the BBBM pre‐screening on the screen‐out rate (the number of individuals who were BBBM negative) and the number of amyloid‐PET and CSF‐positive individuals that were incorrectly screened out were examined. Moreover, we compared the predicted and actual prevalence of amyloid‐PET and CSF positivity and the performance of three different biomarker combinations in the SKYLINE and A4 cohorts: tau phosphorylated at threonine 181 (pTau181) and apolipoprotein E4 protein (ApoE4p); glial fibrillary acidic protein (GFAP) and ApoE4p; and Aβ42/Aβ40 and ApoE4p.

## 2 METHODS

### 2.1 SKYLINE trial design

SKYLINE was a Phase 3, randomized, placebo‐controlled study that terminated early; participants were recruited between April and November 2022.

The primary efficacy measure was the Preclinical Alzheimer's Cognitive Composite‐5 score.^25^ The study aimed to enroll a total of 1200 participants. Treatment duration with either gantenerumab or placebo (randomization 1:1) was planned for 4 years. The target population was participants 60 to 80 years of age, who were cognitively unimpaired (as measured by a Clinical Dementia Rating Global Score of 0 and a Repeatable Battery for the Assessment of Neuropsychological Status Delayed Memory Index of ≥80) and had evidence of elevated cerebral Aβ pathology (as indicated by either positive amyloid‐PET visual read or a pTau181/Aβ42 CSF ratio of >0.04 at the main screening phase).

Study sites had the option to use the BBBM pre‐screening, or alternatively, participants could enter the main screening phase directly without pre‐screening. Participants who chose pre‐screening and were not screened out at this stage due to having a higher likelihood of meeting the amyloid‐based inclusion criteria compared with screened‐out individuals were invited to proceed to the main screening phase. The pre‐screening results were not shared with either the participants or the sites, due to their experimental nature. As a measure to blind the participants from the pre‐screening result, 10% of the participants identified at BBBM pre‐screening as likely to be amyloid negative were invited to proceed to the main screening phase.

**RESEARCH IN CONTEXT**

1. **Systematic review**: Blood‐based biomarker (BBBM) combinations for pre‐screening were selected using samples from the Biomarkers For Identifying Neurodegenerative Disorders Early and Reliably (BioFINDER), Australian Imaging, Biomarker & Lifestyle (AIBL), and Anti‐Amyloid Treatment in Asymptomatic Alzheimer's (A4) studies. The clinical performance of BBBM combinations relating to screen‐out rate and 1‐negative predictive value (1‐NPV) for amyloid positivity was assessed, utilizing data from SKYLINE and A4.
2. **Interpretation**: BBBM pre‐screening led to a screen‐out rate between 38.8% and 46.2% and 1‐NPV ≤2% across both studies, suggesting that BBBM pre‐screening could reduce the number of amyloid‐negative participants undergoing unnecessary, invasive, and costly PET or CSF testing.
3. **Future directions**: Reducing the number of patients undergoing unnecessary PET or CSF testing would alleviate pressure from the health care system and patient burden by avoiding invasive tests unless essential. Future studies should evaluate higher performing BBBMs, such as plasma phosphorylated tau‐217.

**FIGURE 1:**
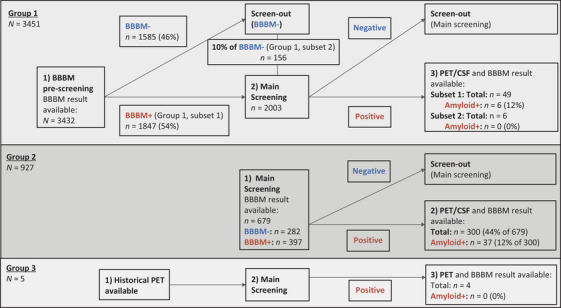
Study population and sample sizes across the three participant SKYLINE groups (Group 1, participants in pre‐screening; Group 2, participants who directly entered the main screening phase; and Group 3, participants who entered the main screening phase with historic PET scans). BBBM (+/−), blood‐based biomarker (positive/negative); CSF, cerebrospinal fluid; PET, positron emission tomography.

As illustrated in Figure 1, SKYLINE screening participants were split into three groups. Group 1: 3432 participants took part in pre‐screening and had blood test results available (subset one had positive results, and subset two had negative results and comprised the 10% of participants that were referred to the main screening phase); some of these individuals underwent confirmatory amyloid testing by PET or CSF. Group 2: participants who entered the main screening phase directly; although these individuals did not take part in the optional pre‐screening, they provided blood samples for BBBM testing as part of the main screening phase, which allowed retrospective evaluation of the pre‐screening algorithm performance in this group. Group 3: participants who entered the main screening phase with historic PET scan results available; a subset of participants also had blood drawn and measured with BBBMs.

The study was conducted in accordance with the International Conference on Harmonization Good Clinical Practice guidelines and the principles of the Declaration of Helsinki. All study participants provided written, informed consent before participating in any pre‐screening and screening assessments and/or intervention activities.

### 2.2 Retrospective cohorts evaluated for this analysis

To select the appropriate biomarker combination for the SKYLINE study, we measured samples and analyzed data from three independent clinical studies, as published previously.^26^ The three clinical studies included: BioFINDER,^22^ AIBL,^23^ and A4.^24^

To optimize an algorithm for implementing a cut‐off rule in this study, we used data from the A4 study,^14^ including the data and samples from individuals who were screen failures for A4. Because A4 was a secondary prevention study, similarly to SKYLINE, the study population was the most comparable out of the available datasets.^24^ BioFINDER and AIBL data were not used in this analysis, beyond previously published data that determined the performance of multiple individual key AD BBBMs in terms of the positive predictive value (PPV) and negative predictive value (NPV) for amyloid positivity and the screen‐out rate.^26^

Acceptance criteria were pre‐defined for clinical performance (screen‐out of amyloid‐PET and CSF‐negative individuals: >40%; 1‐NPV: ≤2%). Additional criteria for biomarker selection and algorithm derivation included robustness of performance with pre‐analytical sample handling variations and measurement error (based on whether an algorithm performs consistently and maintains good clinical performance across studies),^27^ and high analytical sensitivity (based on the lower limit of quantification being less than the cut‐off) to allow precise measurements around the cut‐off.

### 2.3 Samples collected for the BBBMs measured

To select the highest‐performing BBBMs for implementing into the SKYLINE pre‐screening, retrospective measurements were performed using prototype Elecsys^®^ plasma immunoassays on the Cobas^®^ e 601 module (all Roche Diagnostics International Ltd, Rotkreuz, Switzerland). Candidate BBBMs included: Aβ42, Aβ40, ApoE4p, GFAP, neurofilament light chain (NfL), and pTau181.^26^

In the A4 cohort,^28^ it is important to note that the centrifugation of whole blood to separate plasma occurred within 24 h of blood collection for some samples; however, certain biomarkers such as Aβ42 may be susceptible to differences in pre‐analytical sample handling if delays in centrifugation exceeded 2 h after blood collection.^27^, ^29^ This may have impacted clinical utility by showing lower biomarker concentrations than normally measured due to inadequate sample handling.

In SKYLINE, blood samples were collected into 2 × 10 mL K2‐ethylenediaminetetraacetic acid (EDTA) tubes (BD366643, Becton Dickinson). Tubes were inverted 8 to 10 times and were centrifuged within 60 min at 1500 g at 4°C for 10 min; 0.5 mL of blood plasma was aliquoted into 0.5 mL tubes (72.730.005, Sarstedt) and immediately frozen and stored at −70°C. Samples were measured immediately after thawing at the LabCorp Central Laboratory Services (Indianapolis, IN, United States) with different kit lots over a period of 9 months using the prototype plasma immunoassays on the e 601 module for Aβ42, Aβ40, ApoE4p, GFAP, and pTau181; NfL was not measured because the data from the A4 cohort suggested that NfL is not a useful biomarker for the detection of amyloid positivity.

*APOE* genotyping was performed by an in‐house real‐time polymerase chain reaction assay at the LabCorp Greenfields laboratory; blood samples were collected into 3 mL K2‐EDTA tubes (382903664733, Becton Dickinson) and mixed immediately by inverting 8 to 10 times.

### 2.4 Defining/determining amyloid positivity with CSF and/or with PET

In SKYLINE, amyloid positivity based on CSF test results was determined as pTau181/Aβ42 CSF ratio >0.04 using the Elecsys β‐amyloid(1‐42) CSF II and Elecsys Phospho‐Tau (181P) immunoassays on the Cobas e 801 module.^30^ The cut‐off was chosen to increase the PPV with respect to amyloid PET in the study population. In this analysis, 12 mL of CSF was collected in 15 mL tubes (62.554.205, Sarstedt), ensuring that the CSF was not contaminated by blood. Tubes were inverted two to three times and were centrifuged within 30 min at 2000 *g* at 4°C for 10 min; 0.5 mL of supernatant was aliquoted into 0.5 mL tubes (72.730.005, Sarstedt) and immediately frozen at −70°C.

Amyloid positivity based on an amyloid‐PET (including historic PET scans) was determined by a visual read based on the package insert of the three U.S. Food and Drug Administration approved PET tracers: Amyvid,^31^ Vizamyl,^32^ and Neuraceq.^33^

If a PET scan or CSF result was available, the respective result was used to define amyloid positivity. If both results were available, an individual was considered amyloid positive if at least one result from either the PET scan and/or the CSF was positive. An individual was considered amyloid negative if both the PET scan and CSF were negative.

In the A4 study, only PET scan results were available (CSF results were not available), and the study had specific thresholds for amyloid positivity on PET for inclusion into A4.^10^ In this analysis, the visual read was used to determine amyloid positivity for individuals enrolled in A4 and screen failures in A4.

### 2.5 Statistical analysis

Descriptive analyses of baseline characteristics were computed separately for participants in SKYLINE Groups 1, 2, and 3, and for A4 screening participants. Continuous and categoric variables were presented as mean (SD) and *n* (%), respectively.

Biomarkers were presented as mean (SD) and median stratified by amyloid status for SKYLINE Groups 1 and 2 and for A4 participants. Log_10_‐transformed boxplots with jitter points are presented to show the distribution of biomarkers included in the pre‐screening algorithm, stratified by amyloid status in SKYLINE Groups 1 and 2, and for A4 participants. Jitter points are individual data points that are displayed on the plot in addition to the box and whiskers. The jitter aspect refers to adding a slight horizontal spread to the data points to reduce overlap and ensure that individual points are more visible. By preserving the visibility of each data point, the distribution of data can be clearly viewed, even when values are identical or near indistinguishable.

For the screen‐out rate and agreement metrics for the biomarkers included in the pre‐screening algorithm, the number of positives (P; i.e., amyloid positivity), negatives (N; i.e., amyloid negativity), true positives (TPs), true negatives (TNs), false positives (FPs), and false negatives (FNs) were assessed. Prevalence ([TP+FN]/[P+N]), positive percent agreement (PPA; TP/[TP+FN]), negative percent agreement (NPA; TN/[TN+FP]), PPV (TP/[TP+FP]), and 1‐NPV (FN/[TN+FN]) were calculated for SKYLINE Group 2 and for A4 participants. In addition, 95% Wilson confidence intervals were calculated for PPA, NPA, PPV, and 1‐NPV.

PPV, 1‐NPV, and the screen‐out rate were calculated separately for SKYLINE Group 1. In this group, Subsets 1 and 2 were analyzed separately, as only a subset of the individuals with BBBM‐negative results were included in the analysis (as a measure to blind the participants from the pre‐screening result). Therefore, prevalence, PPA, and NPA were not representative and not included in the analysis of Group 1.

For the selected BBBM combination, cumulative predictive risk (PPV and 1‐NPV) was assessed using the R package stats4phc.^34^ Logistic regression models using amyloid status (amyloid negative vs amyloid positive) as the dependent variable and the selected biomarkers as the independent variables were calculated for SKYLINE Group 2 and for A4 participants. Percentiles of the fitted values were plotted against the PPV and 1‐NPV (non‐parametric), and smoothing curves were generated using Constrained Generalized Additive Models.

For all biomarker combinations, areas under the curve (AUCs) were calculated using the R package pROC.^35^ Logistic regression models using amyloid status as the dependent variable and different BBBM combinations as independent variables were estimated. AUCs from each model were calculated and shown in a forest plot. Rounding rules that were applied are detailed in the Supplementary Materials.

For all regression models, Aβ42, Aβ40, GFAP, and pTau181 were log_10_‐transformed, whereas ApoE4p was treated as a binary variable (carrier vs non‐carrier).

## 3 RESULTS

### 3.1 Determination of biomarker combinations for SKYLINE using historic study data

Suitable biomarkers and biomarker combinations were determined based on pre‐defined acceptance criteria (Figure S1). The combination of plasma pTau181 and ApoE4p met all acceptance criteria and was the biomarker combination with the highest clinical performance.^26^, ^36^

Using prevalence‐adjusted A4 cohort data, a pre‐screening algorithm based on two biomarkers, plasma pTau181 and ApoE4p, was optimized and resulted in a predicted screen‐out rate of 45%; a predicted 55% percentage of individuals to be invited to the main screening phase; an expected prevalence increase of 15% to 26%; and a 1‐NPV of 2% for the SKYLINE study, which fulfilled the minimum acceptance criteria of a screen‐out rate of >40% and 1‐NPV of ≤2%. The pre‐screening algorithm had similar performance across cohorts; that is, it showed clinical robustness. The required pre‐analytical sample handling for the prototype plasma pTau181 and ApoE4p immunoassays was feasible for operationalization of sample collection in a global Phase 3 randomized clinical trial; lower limits of quantification were below the cut‐offs for both assays; that is, analytical sensitivity was assumed.^26^, ^36^

### 3.2 Design and implementation of the BBBM screen‐out test in SKYLINE

**FIGURE 2:**
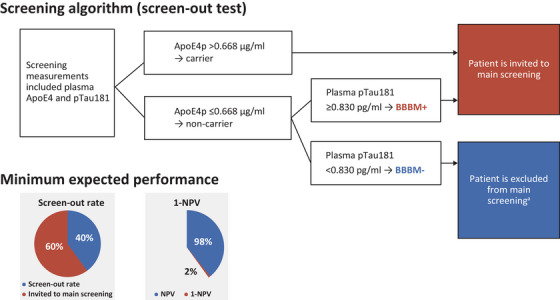
Pre‐screening algorithm and minimum expected performance. ^a^As a measure to blind the participants from the pre‐screening result, 10% of the participants identified at BBBM pre‐screening as likely to be amyloid negative were invited to proceed to the main screening phase. ApoE4, apolipoprotein E4; ApoE4p, apolipoprotein E4 protein; BBBM, blood‐based biomarker; NPV, negative predictive value; pTau181, tau phosphorylated at threonine 181.

The pre‐screening algorithm consisted of a two‐step approach (Figure 2). Participants with ApoE4p concentration >0.668 µg/mL were defined as ApoE4p carriers and were considered BBBM positive. Participants with ApoE4p concentration ≤0.668 µg/mL were defined as ApoE4p non‐carriers and were subjected to pTau181 measurement. ApoE4p non‐carriers with a pTau181 concentration ≥0.830 pg/mL were also considered BBBM positive; ApoE4p non‐carriers with a pTau181 concentration <0.830 pg/mL were considered BBBM negative.

For pre‐screening participants in SKYLINE Group 1, a negative BBBM test result led to exclusion from the main screening phase, whereas a positive test result led to an invitation to the main screening phase. The same definitions for BBBM positive and BBBM negative were applied for participants who entered the main screening phase directly in SKYLINE Group 2; however, results did not lead to exclusion from the main screening phase.

### 3.3 SKYLINE and A4 study population characteristics

Of the 3451 individuals who participated in the SKYLINE pre‐screening, 3432 had available blood test results (Group 1; Figure 1). Of these, 1847 individuals with a positive blood test (Group 1, Subset 1) and 156 with a negative blood test result (Group 1, Subset 2) were invited to the main screening phase; participants who did not meet the inclusion/exclusion criteria of the main screening phase were screened out. In the last step of the main screening phase, confirmatory amyloid results were received for 49 and 6 individuals in SKYLINE Group 1, Subsets 1 and 2, respectively.

A total of 927 individuals entered the main screening phase directly without pre‐screening (Group 2). Of these, 679 individuals had an available blood test result and 300 received confirmatory amyloid results in the last step of the main screening phase. In addition, in Group 3, five individuals entered the main screening phase with available historic PET scan results.

In the A4 cohort, amyloid‐PET results were available for the entire amyloid screening population, except for a small proportion of the first screening visit samples that were measured and were screened out prior to reaching amyloid‐PET assessment.

Overall, the baseline characteristics were well balanced between all SKYLINE participants and the A4 cohort. Small differences were observed in age; in SKYLINE, the mean age (SD) was 69.1 (5.47) years, and in A4, it was 71.5 (4.83) years. Of all SKYLINE and A4 participants, 1428 (32.6%) and 1697 (40.1%), respectively, were male. The majority of participants in both the SKYLINE and A4 studies were White (618 [90.2%] and 3921 [92.6%], respectively) (Table S1).

### 3.4 Performance of the BBBM screen‐out test in SKYLINE and A4

#### 3.4.1 Distribution of plasma pTau181 in SKYLINE and A4

**FIGURE 3:**
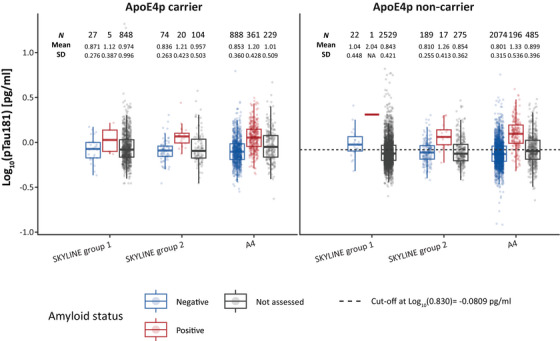
Plasma pTau181 distribution stratified by ApoE4p carrier status and amyloid status. Observations below the plasma pTau181 cut‐off in ApoE4p non‐carriers were screened out. The definition of “not assessed” in SKYLINE is as follows: either due to premature termination of the study, a negative BBBM pre‐screening result, or screen failures during the main screening phase. The definition of “not assessed” in A4 is as follows: screen failures during the screening phase before reaching amyloid assessment. A4, Anti‐Amyloid Treatment in Asymptomatic Alzheimer's Disease; ApoE4p, apolipoprotein E4 protein; pTau181, tau phosphorylated at threonine 181; SD, standard deviation.

The conditional distribution of plasma pTau181 by amyloid status was similar within ApoE4p carrier and non‐carrier groups for both SKYLINE and A4 cohorts (Figure 3). Mean plasma pTau181 levels were higher on average in amyloid‐positive individuals than amyloid‐negative individuals. Mean plasma pTau181 levels in the correctly screened‐out population (ApoE4p non‐carriers who were amyloid negative) were 0.810 pg/mL in SKYLINE Group 2 and 0.801 pg/mL in A4, both of which were lower than the plasma pTau181 cut‐off of 0.830 pg/mL. SKYLINE Group 1 yielded slightly higher plasma pTau181 results in ApoE4p non‐carriers who were amyloid negative (1.04 pg/mL); however, the sample size of this subgroup was small (*n* = 22).

#### 3.4.2 Screen‐out rate and agreement performance results of BBBM pre‐screening test

Of the 3432 individuals with blood test results in SKYLINE Group 1, a total of 1585 had negative pre‐screening results, which resulted in a screen‐out rate of 46.2%. The remaining 1847 (53.8%) had positive pre‐screening results and were invited to the main screening phase (Table 1; Figure 1). The results in SKYLINE Group 2 and in A4 individuals were similar, with confirmed screen‐out rates of 41.5% (282/679) and 38.8% (1644/4233), respectively (Table 1). The 1‐NPV yielded 1.68% in SKYLINE Group 2 and 1.81% in A4 individuals.

**TABLE 1:** Screen‐out rate, prevalence, and agreement results for SKYLINE and A4.

Subset of individuals with available BBBM results				
	SKYLINE Group 1, *N* = 3432		SKYLINE Group 2, *N *= 679	A4, *N* = 4233
BBBM screen‐out rate, *n* (%)	1585 (46.2)		282 (41.5)	1644 (38.8)

Subset of individuals with available BBBM and amyloid results				
	**SKYLINE Group 1, *N *= 55**			
	**Subset 1, *n *= 49**	**Subset 2, *n *= 6**	**SKYLINE Group 2, *N *= 300**	**A4, *N *= 3519**
Prevalence, %	—	—	12.3	15.8
PPA, % (95% CI)	—	—	94.6 (82.3, 98.5)	95.5 (93.5, 96.9)
NPA, % (95% CI)	—	—	44.5 (38.6, 50.5)	45.8 (44.0, 47.6)
PPV, % (95% CI)	12.2 (5.73, 24.2)	—	19.3 (14.2, 25.7)	24.9 (23.1, 26.8)
1‐NPV, % (95% CI)	–	0.0 (0.0, 39.0)	1.68 (0.462, 5.92)	1.81 (1.23, 2.66)

Abbreviations: A4, Anti‐Amyloid Treatment in Asymptomatic Alzheimer's; BBBM (+/−), blood‐based biomarker measurements (positive/negative); CI, confidence interval; NPA, negative percent agreement; NPV, negative predictive value; PPA, positive percent agreement; PPV, positive predictive value.

In SKYLINE Group 2 and in A4 individuals, the prevalence of amyloid positivity after screening increased from 12.3% to 19.3% and from 15.8% to 24.9%, respectively (Table 1). PPA and NPA were similar in SKYLINE Group 2 (94.6% and 44.5%) and in A4 individuals (95.5% and 45.8%), respectively (Table 1). All four individuals from SKYLINE Group 3 had positive pre‐screening results despite being amyloid negative; therefore, PPA was not calculable.

SKYLINE Group 1 Subset 1 (pre‐screening BBBM‐positive participants) had a PPV of 12.2%, and SKYLINE

Group 1 Subset 2 (10% of the pre‐screening BBBM‐negative participants that were sent to main screening) had a 1‐NPV of 0%; although this result outperformed the minimum performance goal of 2%, it is based on a small sample size of only six participants (Table 1).

**FIGURE 4:**
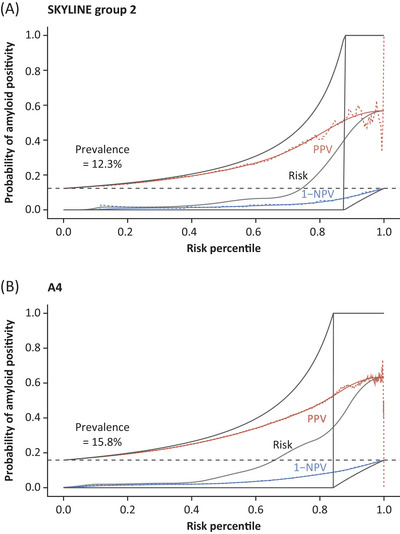
Integrated risk‐profile plot representing the estimated probability of amyloid‐positive individuals in (A) SKYLINE group two and (B) A4 cohort. The probability of individuals being amyloid positive (risk) is black, PPV is in red, and 1‐NPV is in blue for the risk percentiles, that is, the percentiles of the fitted values of the logistic regression model (amyloid status ∼ ApoE4p status + log_10_‐transformed plasma pTau181). Light gray lines indicate optimal PPV, 1‐NPV, and risk curves. Dotted lines indicate observed values, and solid lines are CGAM smoothed curves. The risk percentile corresponds to the screen‐out rate for amyloid positivity. The dashed black line corresponds to the observed prevalence. A4, Anti‐Amyloid Treatment in Asymptomatic Alzheimer's Disease; ApoE4p, apolipoprotein E4 protein; CGAM, Constrained Generalized Additive Models; NPV, negative predictive value; PPV, positive predictive value; pTau181, tau phosphorylated at threonine 181; SD, standard deviation.

Cumulative predictive risk estimates (PPV and 1‐NPV), taking both plasma pTau181 and ApoE4p as predictors, were similar between SKYLINE Group 2 and in A4 individuals. The curve for 1‐NPV was flat over the course of increasing risk percentiles, whereas PPV increased (Figure 4). Non‐parametric and smoothed curves aligned well and were close to the optimal curves, especially in the lower risk percentile (Figure 4).

#### 3.4.3 ApoE4p carrier prevalence in SKYLINE and A4

The ApoE4p carrier prevalence in SKYLINE Groups 1 and 2 was 25.6% and 29.1%, respectively, whereas it was 34.9% in the A4 cohort (Table S1); genetic *APOE*4 carrier prevalence in SKYLINE Groups 1 and 2 was 46.0% and 29.2%, respectively, and 34.8% in A4 individuals. The prevalence of amyloid positivity in ApoE4p non‐carriers in SKYLINE Group 2 and in A4 individuals was 8.65% and 8.63%, respectively, compared with ApoE4p carriers in SKYLINE Group 2 (21.3%) and in A4 individuals (28.9%; Table S2). Concordance between genetic *APOE*4 and ApoE4p was high, with only 2/246 (Group 1), 2/679 (Group 2), and 54/4192 (A4) individuals misclassified by the ApoE4p test (Table S3).

#### 3.4.4 Comparison of BBBM combinations

On average, in SKYLINE Group 1, SKYLINE Group 2, and A4, the levels of plasma pTau181, ApoE4p, and GFAP were lower in amyloid‐negative than in amyloid‐positive individuals, whereas Aβ42 was higher in amyloid‐negative individuals (Table S4). Mean values for Aβ40 differed only slightly between amyloid‐negative and amyloid‐positive individuals and showed no clear trend (SKYLINE Group 1: 299 pg/mL vs 288 pg/mL; SKYLINE Group 2: 293 pg/mL vs 316 pg/mL; A4: 202 pg/mL vs 202 pg/mL; Table S4).

Plasma pTau181 and ApoE4p was the highest‐performing biomarker combination in both SKYLINE Group 2 (AUC: 0.865) and in A4 individuals (AUC: 0.851; Figure S2). The combinations of plasma GFAP and ApoE4p (SKYLINE Group 2 AUC: 0.772; A4 AUC: 0.788), as well as plasma Aβ42/Aβ40 and ApoE4p (SKYLINE Group 2 AUC: 0.774; A4 AUC: 0.755), showed lower discrimination ability versus plasma pTau181 and ApoE4p. Due to the low sample size (six amyloid‐positive individuals) in SKYLINE Group 1, AUC was not assessed.

## 4 DISCUSSION

This analysis demonstrated that BBBM pre‐screening in secondary prevention trials enrolling elderly cognitively unimpaired individuals, such as SKYLINE, was beneficial in reducing the screen failure rate during the main screening phase and alleviating the overall participant and clinical site burden by preventing unnecessary subsequent clinical and biomarker assessments in large volumes of participants. This analysis used historic A4 cohort data to develop a pre‐screening algorithm with pTau181 and ApoE4p for SKYLINE, considering the clinical performance, pre‐analytical sample handling, clinical robustness, and analytical sensitivity.

The performance of BBBM pre‐screening in SKYLINE was in line with the predicted performance based on the A4 cohort.^26^ The predicted screen‐out rate was 45%, and the predicted percentage of individuals to be invited to the main screening phase was 55%. The predicted rates were similar to the actual results, where for SKYLINE Group 1 (*n* = 3432), the screen‐out rate was 46.2%; thus 53.8% were invited to the main screening phase. In addition, the predicted 1‐NPV was similar to the actual result (1−NPV, 2%). The observed prevalence of amyloid positivity was slightly lower in SKYLINE Group 2 (12.3%) than in the A4 study (15.8%), which resulted in a lower PPV after pre‐screening. Moreover, ApoE4p carrier prevalence was slightly lower in SKYLINE Group 2 compared with A4 individuals (29.1% and 34.9%, respectively), as well as amyloid positivity prevalence in ApoE4p carriers (21.3% and 28.9%, respectively). Despite these differences, the pre‐screening algorithm met the clinical performance acceptance criteria (based on a screen‐out rate >40% and 1‐NPV ≤2%) and showed similar performance in terms of screen‐out rate and 1‐NPV. In line with previous studies, ApoE4p showed high concordance with genetic *APOE*4 status^37^, ^38^; however, the prevalence of genetic *APOE*4 in SKYLINE Group 1 was not representative of the underlying population, as only 10% of the BBBM‐negative individuals were genotyped. Plasma pTau181 concentrations in amyloid‐positive and amyloid‐negative individuals were comparable in ApoE4p carriers and non‐carriers. Due to a higher prevalence of amyloid positivity in carriers compared with non‐carriers, inclusion of ApoE4p in the pre‐screening algorithm was pertinent for calibration of probability estimates.

The combination of plasma pTau181 and ApoE4p demonstrated superior clinical performance (SKYLINE AUC: 0.865; A4 AUC: 0.851) when compared to plasma GFAP and ApoE4p (SKYLINE AUC: 0.772; A4 AUC: 0.788) and plasma Aβ42/Aβ40 and ApoE4p (SKYLINE AUC: 0.788; A4 AUC: 0.755), which was in line with the predicted performance.^36^ The logistic regression modelling for the combination of plasma pTau181 and ApoE4p also showed good performance in terms of PPV and 1‐NPV across risk percentiles, especially in the lower risk percentiles, where screen‐out cut‐offs for low prevalence indications are expected.^39^ Plasma pTau181 and ApoE4p, among others, are currently being developed for in vitro device diagnostic use.^40^ However, although plasma pTau181 and ApoE4p performed as expected, future secondary prevention studies could evaluate newer and potentially higher‐performing BBBMs, such as plasma tau phosphorylated at threonine 217 (pTau217), to assess if this would allow for more efficient trial recruitment, with less burden to participants and sites.

With the advent of high‐performing biomarkers like plasma pTau217, the AD field has started using BBBMs not only as rule‐out pre‐screening tests but as rule‐in screening tests or inclusion criteria for secondary prevention studies like TRAILBLAZER‐ALZ3^12^ and TRAILRUNNER‐ALZ3.^41^ This is a promising novel intended use of BBBMs, where the presence of amyloid pathology as an inclusion criterion to enter the study may only be assessed using plasma pTau217, without the need for a confirmatory amyloid‐PET or CSF assessment at the end of the main screening phase. The benefits of rule‐in BBBM testing comprise cost savings and less burden on patients for undergoing, and clinical sites for performing, PET or CSF testing.^17^, ^18^ However, using BBBMs as a rule‐in test does come with the risk that participants ruled‐in by a BBBM result only could still be amyloid negative as per PET or CSF testing, and might not benefit from the pharmacologic intervention, yet may be at risk of adverse events.

This analysis has multiple strengths including that, based on our current knowledge, it is one of the first studies to report BBBM pre‐screening data in a secondary prevention study and provides evidence that the actual performance of BBBM pre‐screening is similar to the predictions made based on historical cohort data. In addition, this analysis measured BBBMs in almost 3500 individuals in only 9 months using a limited number of activated clinical trial sites, which demonstrates the ability to implement time‐saving, cost‐effective, and minimally invasive BBBM pre‐screening tests in individuals and supports the potential for reducing patient and clinical site burden.

This analysis had some limitations, such as the small number of confirmed amyloid‐positive and amyloid‐negative samples by PET or CSF testing due to the premature termination of the study after only 9 months of initiating recruitment. As a result, several endpoints could not be measured in SKYLINE Group 1. Another limitation is that at the time of the study we did not have a high‐performing pTau217 immunoassay at our disposal that had sufficient analytical sensitivity; however, one is now available for use in clinical trials.^42^

In conclusion, this analysis determined that BBBM pre‐screening in secondary prevention trials is beneficial and has the potential to reduce patient burden by decreasing the number of individuals who are required to undergo subsequent costly and invasive amyloid‐PET scans or lumbar punctures as part of CSF testing, and other time‐consuming and burdensome screening assessments.

## CONFLICT OF INTEREST STATEMENT

Alina Bauer and Ekaterina Manuilova are full‐time employees of Roche Diagnostics GmbH, and own stock in F. Hoffmann‐La Roche Ltd. Christina Rabe and Courtney Schiffman are full‐time employees of Genentech, Inc., a member of the Roche group, and own stock in F. Hoffmann‐La Roche Ltd. Fiona Rose is a full‐time employee of Roche Products Ltd and owns stock in F. Hoffmann‐La Roche Ltd. Gesine Respondek, Fabiana Gullotta, and Susanne Ostrowitzki are full‐time employees of F. Hoffmann‐La Roche Ltd and own stock in F. Hoffmann‐La Roche Ltd. Laura Schlieker is a contractor for Roche Diagnostics GmbH and a full‐time employee of Staburo GmbH. Alexander Jethwa is a full‐time employee of Roche Diagnostics GmbH. Isabelle Schrurs is a full‐time employee of Roche Diagnostics International Ltd. Tobias Bittner is a full‐time employee of F. Hoffmann‐La Roche Ltd and Genentech, Inc., a member of the Roche group, and owns stock in F. Hoffmann‐La Roche Ltd. Author disclosures are available in the Supporting Information.

## CONSENT STATEMENT

All study participants provided written, informed consent before participating in any pre‐screening and screening assessments and/or intervention activities in the SKYLINE and Anti‐Amyloid Treatment in Asymptomatic Alzheimer's (A4) studies.

## Supporting information



Supporting Information

Supporting Information
