# Supplementary material for: Blood‐based pre‐screening in the SKYLINE secondary prevention Ph3 gantenerumab study
Source: Alzheimers Dement. 2025 Oct 14;21(10):e70676. doi: 10.1002/alz.70676 (PMC12519518; doi:10.1002/alz.70676)
Supplement: Supplementary file 1 — Supporting Information [file ALZ-21-e70676-s002.docx]

**Supplementary Methods**

**Rounding rules:**

1. "Round half away from zero" should be applied for rounding.
2. The number of decimal places of the rounded number depends on the magnitude of the absolute value of the original number:
   1. Numbers ≥100 are rounded to the nearest integer
   2. Numbers ≥0.0001 and <100 are rounded to three significant digits
   3. Numbers <0.0001, are rounded to six decimal places
   4. Results that are smaller than 0.000001 are reported as “<0.000001”
3. Exceptions:
   1. *P*-values of statistical significance tests are rounded to four decimal places
   2. *P*-values below .0001 are reported as “< .0001”
   3. The following numbers are not rounded:
      1. Integer values (e.g., sample size)
      2. Intermediate results
      3. Assigned values
      4. Raw data (e.g., counts).

**Supplementary Results**

**FIGURE S1** Acceptance criteria for candidate BBBMs.**
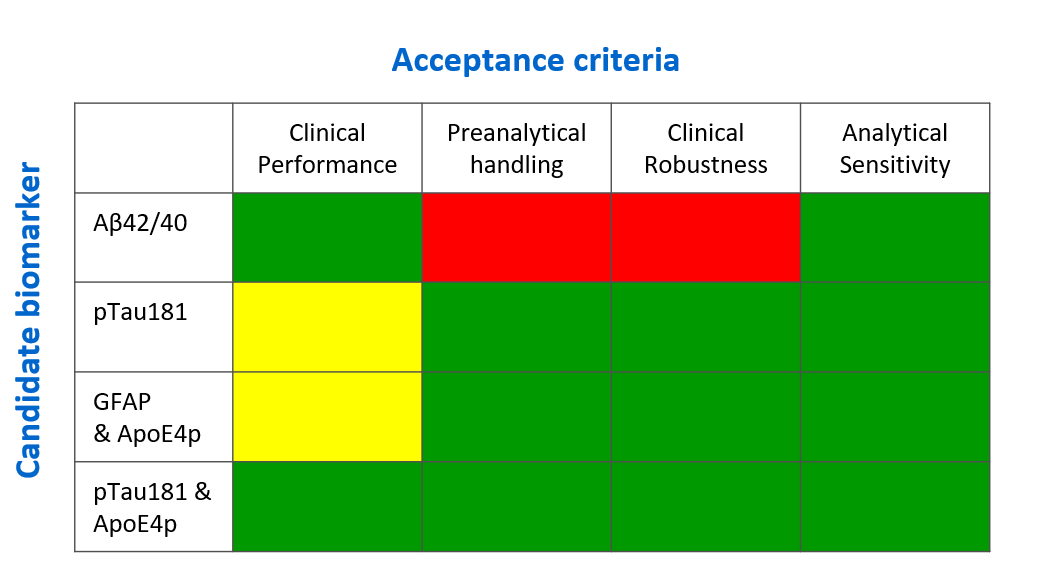
**

Green boxes are fulfilled; yellow are borderline; and red are not fulfilled. Clinical performance was defined as screen-out of >40% at 1-NPV ≤2%. Preanalytical handling was defined as the ability to operationalize sample collection. Clinical robustness was based on whether an algorithm performs consistently and maintains good clinical performance across studies. Analytical sensitivity was defined as LLoQ of assay < cutoff. Aβ, amyloid-beta; ApoE4p, apolipoprotein E4 protein; BBBM, blood-based biomarker; GFAP, glial fibrillary acidic protein; LLoQ, lower limit of quantification; NPV, negative predictive value; pTau181, tau phosphorylated at threonine 181.

**FIGURE S2** Area under the curve-receiver operating characteristic analyses (logistic regression model) for different BBBMs based on all participants with an amyloid status.

**
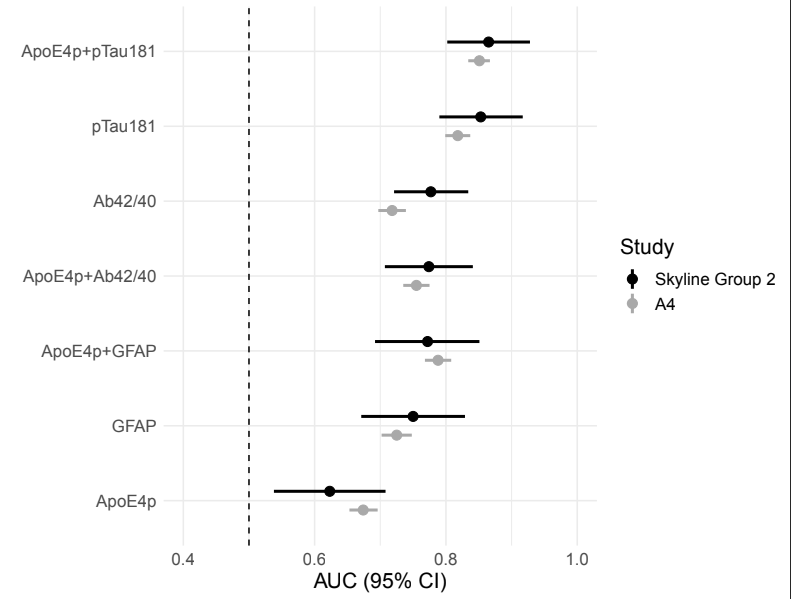
**

A4, Anti-Amyloid Treatment in Asymptomatic Alzheimer’s Disease; Ab, amyloid-beta; AUC, area under the curve; ApoE4p, apolipoprotein E4 protein; BBBMs, blood-based biomarkers; CI, confidence interval; GFAP, glial fibrillary acidic protein; pTau181, tau phosphorylated at threonine 181.

**TABLE S1**: Baseline demographics and characteristics of the SKYLINE and A4 study populations.

|  | **SKYLINE Total *N*= 4383** | **SKYLINE group one* *N*= 3451** | **SKYLINE group two *N*= 927** | **SKYLINE group three *N*= 5** | **A4 with ≥1 biomarker result *N*= 4235** |
| --- | --- | --- | --- | --- | --- |
| Mean age, years (SD) Missing | 69.1 (5.47) 0 | 68.9 (5.47) 0 | 69.7 (5.46) 0 | 69.0 (6.78) 0 | 71.5 (4.83) 0 |
| Sex, *n* (%) Male Female Missing | 1428 (32.6) 2955 (67.4) 0 | 1122 (32.5) 2329 (67.5) 0 | 305 (32.9) 622 (67.1) 0 | 1 (20.0) 4 (80.0) 0 | 1697 (40.1) 2538 (59.9) 0 |
| Race, *n* (%) Black/African American American Indian/Alaska  Asian White Native Hawaiian/Pacific Islander Multiple Not reported Unknown Missing | 57 (8.32) 0 (0.0) 0 (0.0) 618 (90.2) 0 (0.0) 0 (0.0) 7 (1.02) 3 (0.438) 3698 | 7 (5.30) 0 (0.0) 0 (0.0) 123 (93.2) 0 (0.0) 0 (0.0) 2 (1.52) 0 (0.0) 3319 | 49 (8.93) 0 (0.0) 0 (0.0) 492 (89.6) 0 (0.0) 0 (0.0) 5 (0.911) 3 (0.546) 378 | 1 (25.0) 0 (0.0) 0 (0.0) 3 (75.0) 0 (0.0) 0 (0.0) 0 (0.0)) 0 (0.0) 1 | 169 (3.99) 10 (0.236) 70 (1.65) 3921 (92.6) 3 (0.0708) 33 (0.779) 0 (0.0) 29 (0.685) 0 |
| Genetic *APOE4* carrier, *n* (%) Non-carrier Heterozygous carrier Homozygous carrier Missing | 648 (66.3) 298 (30.5) 31 (3.17) 3406 | 133 (54.1) 102 (41.5) 11 (4.47) 3205 | 515 (70.8) 194 (26.7) 18 (2.48) 200 | 0 (0.0) 2 (50.0) 2 (50.0) 1 | 2734 (65.2) 1324 (31.6) 136 (3.24) 41 |
| Plasma ApoE4p carrier^†^, *n* (%) Non-carrier Carrier Missing | 3036 (73.7) 1083 (26.3) 264 | 2552 (74.4) 880 (25.6) 19 | 484 (70.9) 199 (29.1) 244 | 0 (0.0) 4 (100) 1 | 2755 (65.1) 1478 (34.9) 2 |
| Amyloid positivity, *n* (%) Negative Positive Missing | 340 (88.1) 46 (11.9) 3997 | 49 (89.1) 6 (10.9) 3396 | 286 (87.7) 40 (12.3) 601 | 5 (100) 0 (0.0) 0 | 2964 (84.2) 557 (15.8) 714 |
| CSF positivity^‡^, *n* (%) Negative Positive Missing | 105 (90.5) 11 (9.48) 4267 | 7 (100.0) 0 (0.0) 3444 | 97 (89.8) 11 (10.2) 819 | 1 (100.0) 0 (0.0) 4 | 0 (0.0) 0 (0.0) 4235 |
| PET positivity, *n* (%) Negative Positive Missing | 311 (88.4) 41 (11.6) 4031 | 44 (88.0) 6 (12.0) 3,401 | 262 (88.2) 35 (11.8) 630 | 5 (100.0) 0 (0.0) 0 | 2964 (84.2) 557 (15.8) 714 |

Abbreviations: A4, Anti-Amyloid Treatment in Asymptomatic Alzheimer’s Disease; *APOE4*, apolipoprotein E4 gene; ApoE4p, apolipoprotein E4 protein; Aβ, amyloid-beta; BBBM, blood-based biomarkers; CSF, cerebrospinal fluid; PET, positron emission tomography; pTau181, tau phosphorylated at threonine 181; SD, standard deviation.
*Note that only 10% of the participants identified at BBBM pre-screening as amyloid negative were invited to proceed to the main screening phase, where amyloid positivity was assessed.
^†^Non-carrier: ApoE4p ≤0.668 μg/ml; carrier: ApoE4p >0.668 μg/ml.
^‡^Positive: CSF pTau181/Aβ_42_ >0.04; negative: otherwise.

.

**TABLE S2** Observed amyloid positivity prevalence by plasma ApoE4p status in A4 individuals and SKYLINE group one and two (subset of participants with available plasma ApoE4p and amyloid status).

| **Amyloid positivity, *n* (%)** | **Plasma ApoE4p  non-carrier** | **Plasma ApoE4p  carrier** | **Total (100%)** |
| --- | --- | --- | --- |
| Amyloid positive A4 SKYLINE group one  SKYLINE group two | 196 (8.63) 1 (4.35) 18 (8.65) | 361 (28.9) 5 (15.6) 20 (21.3) | 557 6 38 |
| Amyloid negative A4 SKYLINE group one SKYLINE group two | 2074 (91.4) 22 (95.7) 190 (91.3) | 888 (71.1) 27 (84.4) 74 (78.7) | 2962 49 264 |
| Total (100%) A4 SKYLINE group one  SKYLINE group two | 2270 (100.0) 23 (100.0) 208 (100.0) | 1249 (100.0) 32 (100.0) 94 (100.0) | 3519 55 302 |

Abbreviations: A4, Anti-Amyloid Treatment in Asymptomatic Alzheimer’s Disease;
ApoE4p, apolipoprotein E4 protein.

**TABLE S3** Concordance between plasma ApoE4p and genetic *APOE4* results in A4 and SKYLINE group one and two (subset of participants with available plasma ApoE4p and genetic *APOE4* results).

| **Genetic *APOE4*, *n* %** | **Plasma ApoE4p non-carrier** | **Plasma ApoE4p carrier** | **Total (100%)** |
| --- | --- | --- | --- |
| **Genetic non-carrier** A4 SKYLINE group one  SKYLINE group two | 2703 (99.1) 131 (100.0) 479 (99.6) | 29 (1.98) 2 (1.74) 0 (0.00) | 2732 133 479 |
| **Genetic carrier** A4 SKYLINE group one SKYLINE group two | 25 (0.916) 0 (0.000) 2 (0.416) | 1435 (98.0) 113 (98.3) 198 (100.0) | 1460 113 200 |
| **Total (100%)** A4 SKYLINE group one SKYLINE group two | 2728 (100) 131 (100) 481 (100) | 1464 (10) 115 (100) 198 (100) | 4192 246 679 |

Abbreviations: A4, Anti-Amyloid Treatment in Asymptomatic Alzheimer’s Disease;
APOE4, apolipoprotein E4 gene; ApoE4p, apolipoprotein E4 protein.

**Table S4**: Biomarker distributions stratified by amyloid status in SKYLINE group one and two and in A4 individuals (subset of participants with at least one available biomarker).

|  | **SKYLINE group one** | | | **SKYLINE group two** | | | **A4** | | |
| --- | --- | --- | --- | --- | --- | --- | --- | --- | --- |
|  | **Total *N*=3451** | **Amyloid positive *N*=6** | **Amyloid negative *N*=49** | **Total *N*=702** | **Amyloid  positive *N*=38** | **Amyloid negative *N*=277** | **Total *N*=4235** | **Amyloid positive *N*=557** | **Amyloid negative *N*=2964** |
| ApoE4p (μg/ml) *n* Mean (SD) Median Missing | 3423 1.23 (2.39) 0.0200 19 | 6 3.78 (3.07) 3.23 0 | 49 3.01 (3.63) 2.69 0 | 683 1.49 (2.87) 0.0200 19 | 38 2.77 (3.48) 2.22 0 | 264 1.33 (2.41) 0.0200 13 | 4233 2.50 (4.13) 0.0879 2 | 557 4.62 (4.71) 4.26 0 | 2962 2.14 (3.89) 0.0879 2 |
| pTau181 (pg/ml) *n* Mean (SD) Median Missing | 3432 0.878 (0.618) 0.770 19 | 6 1.28 (0.510) 1.22 0 | 49 0.949 (0.370) 0.888 0 | 679 0.876 (0.368) 0.785 23 | 37 1.24 (0.413) 1.16 1 | 263 0.818 (0.257) 0.780 14 | 4233 0.893 (0.399) 0.800 2 | 557 1.25 (0.472) 1.15 0 | 2962 0.816 (0.330) 0.753 2 |
| Aβ_40_ (pg/ml) *n* Mean (SD) Median Missing | 3415 288 (54.5) 290 36 | 6 288 (38.7) 285 0 | 49 299 (54.6) 290 0 | 680 292 (61.0) 290 22 | 38 316 (50.4) 320 0 | 264 293 (50.9) 295 13 | 4235 204 (152) 209 0 | 557 202 (58.5) 211 0 | 2964 202 (55.0) 207 0 |
| Aβ_42_ (pg/ml) *n* Mean (SD) Median Missing | 3424 33.7 (8.42) 33.3 27 | 6 28.2 (8.91) 28.3 0 | 49 35.3 (9.15) 34.7 0 | 685 32.0 (7.69) 32.4 17 | 37 30.1 (5.41) 30.6 1 | 270 32.6 (7.40) 32.7 7 | 4235 20.9 (7.41) 21.0 0 | 557 18.6 (6.28) 19.4 0 | 2964 21.2 (7.18) 21.5 0 |
| Aβ_42_/_40_ *n* Mean (SD) Median Missing | 3413 0.118 (0.0257) 0.118 38 | 6 0.0977 (0.0265) 0.105 0 | 49 0.120 (0.0299) 0.117 0 | 674 0.112 (0.0361) 0.109 28 | 37 0.0953 (0.00971) 0.0964 1 | 261 0.113 (0.0348) 0.110 16 | 4235 0.120 (0.497) 0.103 0 | 557 0.104 (0.120) 0.0921 0 | 2964 0.124 (0.588) 0.105 0 |
| GFAP (pg/ml) *n* Mean (SD) Median Missing | 3422 96.1 (79.9) 82.0 29 | 6 141 (62.7) 122 0 | 49 95.9 (62.6) 83.0 0 | 680 108 (58.9) 94.0 22 | 38 158 (65.3) 145 0 | 263 107 (62.2) 94.0 14 | 4235 106 (55.7) 95.0 0 | 557 141 (62.8) 131 0 | 2964 99.8 (51.5) 90.0 0 |

Abbreviations: A4, Anti-Amyloid Treatment in Asymptomatic Alzheimer’s Disease; Aβ, amyloid-beta; ApoE4p, apolipoprotein E4 protein;
GFAP, glial fibrillary acidic protein, pTau181, tau phosphorylated at threonine 181; SD, standard deviation.
